# Extending the clinical capabilities of short- and long-lived positron-emitting radionuclides through high sensitivity PET/CT

**DOI:** 10.1186/s40644-022-00507-w

**Published:** 2022-12-16

**Authors:** Joyce van Sluis, Ronald Borra, Charalampos Tsoumpas, Johannes H. van Snick, Mostafa Roya, Dik ten Hove, Adrienne H. Brouwers, Adriaan A. Lammertsma, Walter Noordzij, Rudi A.J.O. Dierckx, Riemer H.J.A. Slart, Andor W.J.M. Glaudemans

**Affiliations:** Medical Imaging Center, Department of Nuclear Medicine and Molecular Imaging, University of Groningen, University Medical Center Groningen, Hanzeplein 1, 9713GZ Groningen, the Netherlands

## Abstract

This review describes the main benefits of using long axial field of view (LAFOV) PET in clinical applications. As LAFOV PET is the latest development in PET instrumentation, many studies are ongoing that explore the potentials of these systems, which are characterized by ultra-high sensitivity. This review not only provides an overview of the published clinical applications using LAFOV PET so far, but also provides insight in clinical applications that are currently under investigation. Apart from the straightforward reduction in acquisition times or administered amount of radiotracer, LAFOV PET also allows for other clinical applications that to date were mostly limited to research, e.g., dual tracer imaging, whole body dynamic PET imaging, omission of CT in serial PET acquisition for repeat imaging, and studying molecular interactions between organ systems. It is expected that this generation of PET systems will significantly advance the field of nuclear medicine and molecular imaging.

## Introduction

Since the 1970s, when the first PET systems were built, there has been a significant evolution in PET technology. Over the last couple of years, progress in development of detector technology from photomultiplier tubes to silicon-based photomultiplier (SiPM) detector elements has led to the development of commercially available digital PET/CT scanners. With the introduction of SiPM-based digital PET/CT systems, time-of-flight (TOF) improved to a range of 210–400 ps and sensitivity increased up to 20 kcps/MBq [1, 2]. Because of the compact size of SiPM-based detector elements, crystals of less than 4 × 4 mm in cross section could be implemented allowing for improved spatial resolution. This increased spatial resolution, combined with higher sensitivity and improved TOF resolution, has resulted in better noise properties. These improved physical performance characteristics subsequently translated into improved image quality and a more efficient use of digital PET systems in daily clinical practice.

The latest improvement in PET system technology is the development of long axial field of view (LAFOV), or so-called “total-body”, PET/CT systems. Also equipped with SiPM-based detectors, these systems surround the patient with many more detectors in the axial direction resulting in two major improvements [3]:

1. higher detection efficiency as more photon pairs are captured.
2. one bed position covers all relevant organs of interest simultaneously.

To date, three LAFOV systems have been introduced: the uEXPLORER (United Imaging Healthcare America) [4] with a 194-cm-long axial FOV; the Siemens Biograph Vision Quadra PET/CT (Siemens Healthineers) [5] with a 106-cm-long axial FOV; and the PennPET Explorer (University of Pennsylvania) [6, 7] with a 64-cm-long axial FOV (the UPENN project is a non-regulatory approved academic research project).

This review will elaborate further on the advantages of LAFOV and provide an overview of novel clinical applications made possible by the use of short- and long-lived positron-emitting tracers within the context of LAFOV PET. Furthermore, the typical challenges encountered when implementing and validating a LAFOV system for clinical use will be discussed.

## General advantages of LAFOV Pet

The main characteristic of novel LAFOV PET/CT scanners is the possibility to cover the whole body (uEXPLORER) or the most important part of the body within an oncological setting (from skull vertex to mid-thigh) including all relevant organs (Quadra) in one single bed position. This provides four major advantages over PET/CT systems with a conventional FOV:

1. Decreased acquisition time and the possibility to implement fast or ultra-fast acquisition protocols, thereby reducing motion artifacts and the need for sedation in e.g. children, which is particularly useful for scanning “difficult” patients, such as patients admitted to the Intensive Care Unit (ICU), severely debilitated patients, claustrophobic patients, or patients who cannot lay still due to neurological disorders or extreme pain.
2. The possibility to reduce administered activity of radiopharmaceuticals with, a corresponding reduction in radiation exposure, which can be of invaluable importance in small children or babies, and in pregnant women.
3. The improved spatial resolution and increased sensitivity may lead to higher diagnostic accuracy, especially in those cases which led to false-negative scan results on conventional FOV scanners, due to e.g., a very low grade tumor or a chronic low-grade infection site.
4. The possibility to perform whole body dynamic PET imaging, without the need for arterial blood sampling, and including all relevant organs in the large FOV, providing the possibility to look at all relevant organs and possible (tumor) lesion simultaneously.

## Specific benefits for short-lived radionuclides

The short half-life of commonly used radionuclides such as ^68^Ga (half-life 67.71 min), ^11^C (half-life 20.39 min) and ^15^O (most used as ^15^O-H_2_O – half-life 2.04 min) can be used in the context of LAFOV PET – benefiting of the high sensitivity and large coverage in several clinically relevant ways.

Obvious benefits are, e.g., to increase the number of performed scans per produced batch/production run of these tracers because of the ability to inject a lower dose, scan faster, and inject at a timepoint longer after the production run – as the very high sensitivity of LAFOV PET systems will still allow for good quality scans. As these short lived tracers can be highly costly to produce, improving the utilization per production run in a clinical or research context may also be highly relevant from a financial perspective.

Furthermore, an obvious benefit of LAFOV combined with ultra-short half-life tracers such as ^15^O-H_2_O is that it potentially allows for evaluating tracer uptake throughout the body before it decays beyond detectability, which currently requires multiple injections of the same tracer for multiple bed positions in case of conventional FOV systems. Capturing tracer dynamics with a single bed position covering alle relevant organs of interest is another benefit for short half-life tracers which, for example, brings whole-body ^15^O-H_2_O perfusion measurements with a single injection within reach, which could be highly relevant in infection/inflammation, cardiovascular and oncological imaging. A practical example of LAFOV PET/CT enabling late imaging of a short-lived radiotracer within the context of recurrent prostate cancer imaging using ^68^Ga-PSMA is provided by Alberts et al., who compared late imaging (4 h post injection (p.i.)) with standard imaging (1 h p.i.), with the aim of improving lesion to background and contrast [8]. This study showed improved TBR and SNR for late acquisitions, and suggests that late imaging might be the preferred approach on LAFOV PET/CT systems in this specific context.

Also, and possibly clinically one of the most relevant benefits, LAFOV allows for combining a short-lived and longer-lived radioisotope scan within the same scan session or on the same day – while still staying within clinically acceptable acquisition time and cumulative patient dose limits. A good example of this is a recent study by Alberts et al. combining ^68^Ga-PSMA with ^18^F-FDG in a dual-tracer same-day imaging protocol in patients referred for ^177^Lu-PSMA-radioligand therapy. In this protocol patients were scanned with the Quadra LAFOV PET/CT scanner 1 h postinjection of a standard dose of ^68^Ga-PSMA (150 MBq) and an additional low-dose (40 MBq) ^18^F-FDG scan one hour thereafter – with the combined protocol identifying lesions with low ^68^Ga-PSMA but high ^18^F-FDG avidity in 1 out of 14 (7%) patients [9].

## Specific benefits for long-lived radionuclides

The most commonly used long-lived radionuclide is Zirconium-89 (^89^Zr). Advantages of ^89^Zr such as the long half-life of 78.4 h, matching the pharmacokinetic behavior of antibodies, and good in vivo stability, make it suitable for labeling monoclonal antibodies (mAbs) [10]. ^89^Zr-immunoPET can provide whole body information on (tumor) target expression [11]. Another long-lived radiotracer of interest is ^124^I, which is used for the detection of differentiated thyroid cancer [12]. However, both tracers have a low positron abundance (23%, as opposed to ^18^F with an abundance of 96%) [10, 12]. Hence, PET imaging suffers from a low signal to noise ratio when acquiring PET images on conventional FOV PET/CT systems. In addition, the long physical half-life limits the amount of radiotracer activity that can be administered to keep radiation exposure within acceptable limits [13].

Currently, immunoPET is used almost exclusively in research settings in oncological patients with a relatively shorter life expectancy, because of the high mean effective doses (ranging from 0.36 to 0.66 mSv/MBq) associated with ^89^Zr-labeled mAbs [14]. Administering a standard amount of 37 MBq of ^89^Zr activity results in a radiation exposure of up to 25 mSv.

**Fig. 1:**
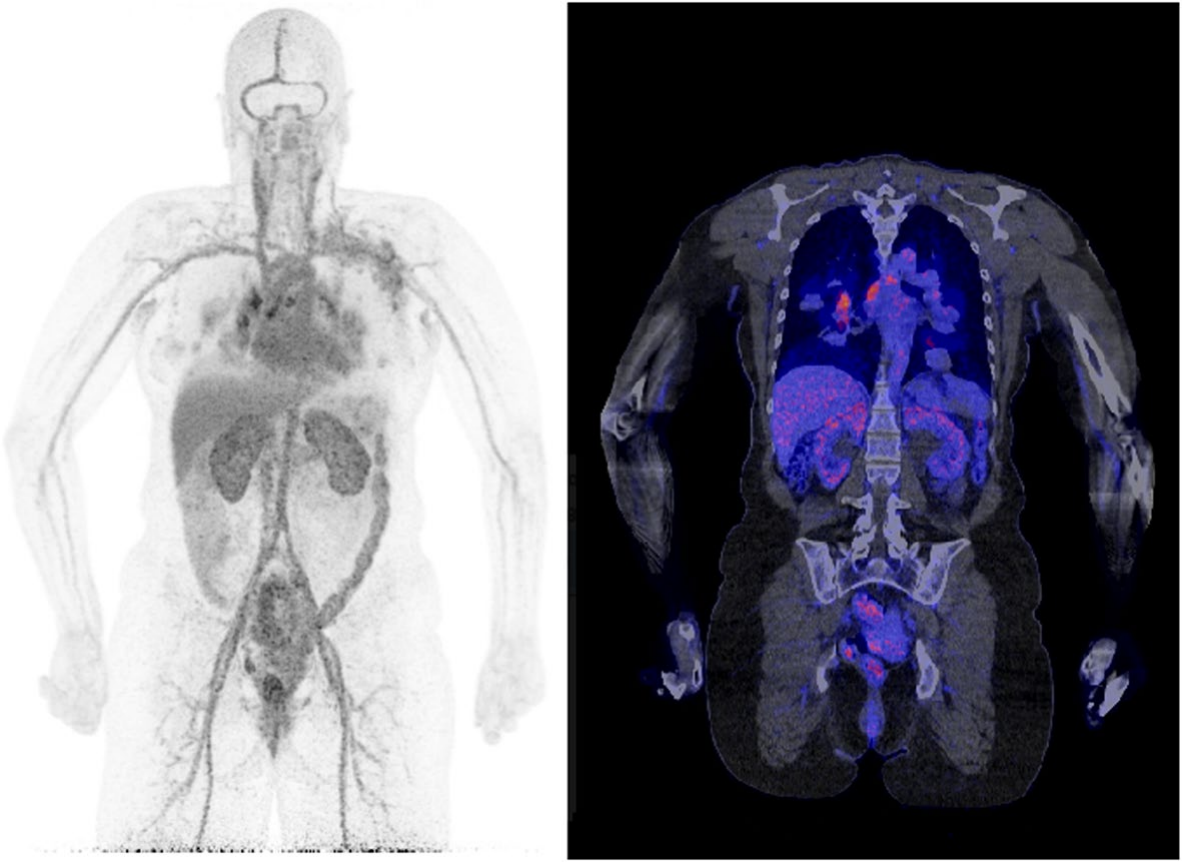
Thirty minutes illustrative example patient ^89^Zr-trastuzumab LAFOV PET/CT images of a 46-year-old female patient with metastasized HER-2 positive breast cancer. The images show multiple mediastinal lymph node metastases

LAFOV PET opens up several possibilities in the field of PET-imaging with long-lived tracers. The increased sensitivity leads to a better signal-to-noise ratio (Fig. 1). Furthermore, it opens the possibility of lowering the amount of administered radioactive ^89^Zr activity so that radiation exposures below 10 mSv become possible. This could allow for the use of immunoPET not only as a last resort in oncology, but also in (younger) patients with benign or inflammatory disorders, first in a research setting, and in the future maybe also in a routine clinical setting. For ^124^I imaging, improved image quality could lead to improved lesion detectability in thyroid cancer.

The higher sensitivity of LAFOV may also enable prolonged uptake time which is expected to result in an improved (tumor) lesion-to-background ratio. Combining delayed imaging with novel radioactive agents allows extended study of in vivo biology [15]. Furthermore, labelled immune cells together with a LAFOV PET/CT system, capturing all relevant organ tissues of interest simultaneously could be used to study crosstalk between different organ systems, e.g., organ axes or the human connectome.

## Overview of current clinical applications

Clinical experiences with LAFOV PET systems have been compared with analog and digital conventional FOV PET systems. As well as experiences regarding clinical optimization using LAFOV PET alone. As the PennPET explorer is still in its prototype stage, this subsection will focus on existing comparison studies between commercially available conventional FOV and LAFOV PET systems, published up to November 2022.

Alberts et al. [16] reported the first clinical experiences in using a LAFOV Biograph Vision Quadra PET/CT with respect to a conventional FOV digital Biograph Vision PET/CT (Siemens Healthineers). A head-to-head comparison was performed between image quality of the Vision Quadra (sensitivity of 174 cps/kBq and a TOF performance of 219 ps [17]) with that of the Vision system (sensitivity of 16.4 cps/kBq and a TOF performance of 210 ps [18]). This intra-individual head-to-head comparison was performed in 44 patients referred for routine oncological ^18^F-FDG, ^18^F-PSMA-1007, and ^68^Ga-DOTA-TOC examinations. The comparison showed improved lesion detectability, reduced image noise levels, and visually improved visually image quality, all in favor of the Vision Quadra. In addition, it was concluded that LAFOV images of equivalent quality to images acquired for $\sim$ 16 min on the conventional digital FOV system can be obtained in 2 min. This reduction in scan duration was found to be interchangeable with reducing the amount of administered radiotracer activity.

This potential to reduce scan duration for oncological ^18^F-FDG imaging using an LAFOV Vision Quadra PET/CT was confirmed by Van Sluis et al. [19] in a study exploring European Association of Nuclear Medicine Research Ltd. (EARL) compliance, who also showed that semiquantitative accuracy was maintained for reduced scan durations. For EARL standard compliant acquisition and reconstruction protocols, scan durations could even be reduced to 1 min.

Another, previously mentioned study by Alberts et al. [8] on an LAFOV Vision Quadra showed that late time point acquisitions using ^68^Ga-PSMA-11 at 4 h p.i. were not only feasible, but even produced improved image quality compared with conventional FOV systems.

With respect to long-lived radionuclides, immunoPET with ^89^Zr-labeled mAbs showed a remarkable improvement in image quality of patients scanned 4 days post p.i. [20]. In this study, images were obtained on an LAFOV Vision Quadra and on either a conventional digital Vision PET/CT or an analog mCT PET/CT (Siemens Healthineers) for a direct visual comparison of image quality. Images as short as 3 min obtained on the LAFOV system showed comparable image quality as 32 and 45 min acquisition times on conventional FOV Vision and mCT systems, respectively.

The first clinical experiences with the uEXPLORER have been described by Badawi et al. in 5 patients undergoing different acquisition protocols including dynamic ^18^F-FDG total-body imaging [21]. The uEXPLORER, with a measured system sensitivity of 174 cps/kBq and a TOF performance of 505 ps [4], was found to image better, faster (as fast as 18.75 s), at later time points after injection (e.g., up to 10 h after injection) or with lower amounts of administered radiotracer (e.g., with only 5.7 MBq injected ^18^F-FDG activity) compared with conventional PET/CT imaging. Furthermore, it was shown that the system was able to acquire total-body dynamic imaging data with high temporal resolution.

Regarding evaluation of pediatric malignancies with half-dose ^18^F-FDG protocols (1.85 MBq/kg), Chen et al. [22] found that acquisition times as short as 1 min resulted in images of adequate diagnostic image quality with sufficient lesion detectability [23] which is imperative for pediatric patients undergoing frequent PET imaging during disease management. Furthermore, ultra-fast 30 s ^18^F-FDG total-body PET imaging in 88 oncologic patients (3.7 MBq/kg) resulted in images with sufficient quality to meet clinical diagnostic requirements [24], although a clear reduction in image quality was seen for the 30 s images compared with the 300 s images. This study concluded, that for patients unable to lie still for 5 min, a 30 s scan would still enable clinical diagnosis.

In addition, one study examined the pathophysiological changes in CD8 + T cell distribution in recovering COVID-19 patients, using a ^89^Zr-labeled minibody [25]. When injecting < 37 MBq of ^89^Zr-labeled mAb, high quality images were obtained with the possibility of deriving parametric Patlak images. This study highlighted that it is feasible to follow in vivo migration of T-cells using LAFOV PET, which allows for exploring functional aspects such as vaccine responses, but which may also be important for immunological research in general [25].

Finally, improvements in calculated liver dosimetry using the LAFOV uEXPLORER versus the conventional analog mCT PET/CT in transarterial radioembolization of liver tumors with ^90^Y microspheres was investigated in two patients by Costa et al. [20]. Even though images obtained using LAFOV PET showed increased signal to noise ratio, they found that the total absorbed dose in the liver showed excellent agreement regardless of PET/CT system, but that there were differences of up to 60% when comparing liver segment doses [26]. The improved signal to noise ratio obtained using LAFOV PET, especially in lower count regions of interest, is expected to improve dosimetry calculations which warrants further investigations.

## Clinical indications

### Oncology

The advantages of a highly sensitive LAFOV PET system over conventional PET systems in oncology can be divided in three major areas: reduction in administered activity or faster scanning in critically ill patients, prolonged time point imaging, and quantification of uptake as a marker of total tumor load. Especially in oncology, early response assessment is pivotal in distinguishing responders from non-responders. Reduction in administered activity opens the possibility to perform these response assessments more frequently. Furthermore, it opens op opportunities to perform scans with multiple different tracers to more accurately map the status of the disease. In case a patient is not responding to the treatment, a switch to an alternative treatment line can be made more swiftly, potentially resulting in less treatment related toxicity [27]. As such, it may contribute to better personalized treatment strategies, eventually leading to increased survival in this patient group. Due to better and more effective treatment strategies, survival from any malignancy has improved in the last decades [28]. As a result, patients are scanned more often during their (extended) follow-up. Reduction in administered activity during these follow-up investigations is pivotal for keeping the cumulative radiation burden within acceptable ranges. This also accounts for repeat imaging necessary in (younger) Hodgkin lymphoma or melanoma patients with a relatively high life expectancy [29].

In addition, LAFOV PET/CT scanning will contribute to a better understanding of the biodistribution of newly developed tracers, since different organ axes can be visualized and studied in one image. The addition of dynamically acquired kinetic information can play a role in the assessment of therapeutic efficacy [30]. Furthermore, dynamic acquisition helps to better quantify tracer uptake in tumor lesions, free from confounding signals such as non-specific uptake, as well as (interinstitutional) comparison of tracer uptake and lesion-to-normal tissues ratio of different tracers for the same application.

**Fig. 2:**
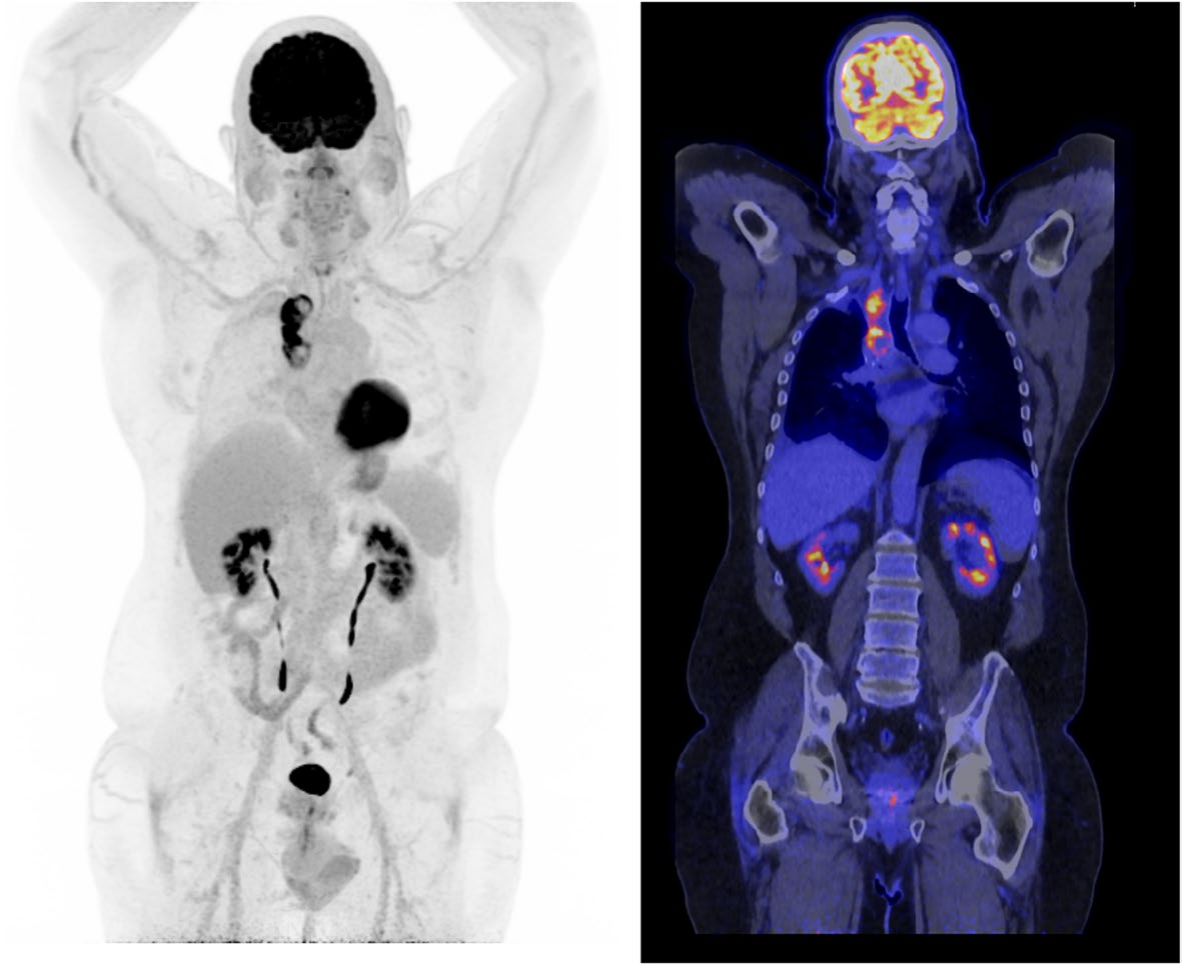
Illustrative patient example of a 10 min ^18^F-FDG LAFOV PET/CT of a 65-year-old male patient with a residual tumor lesion after right upper lobectomy

For ^18^F-FDG, the most commonly used radiopharmaceutical in clinical practice, the main advantages of an LAFOV PET/CT system is the reduction in scanning time which may lead to a higher patient throughput. It is not expected that the diagnostic accuracy, which is already high for most oncological diseases, will further increase (Fig. 2).

The published studies so far in this field predominantly compare the diagnostic performance between standard and reduced scan acquisition times. In a study on 78 patients with hepatic tumors, no significant differences were seen in the number of detected hepatic lesions between standard (15 min) and fast (2 min) scans [31].

**Fig. 3:**
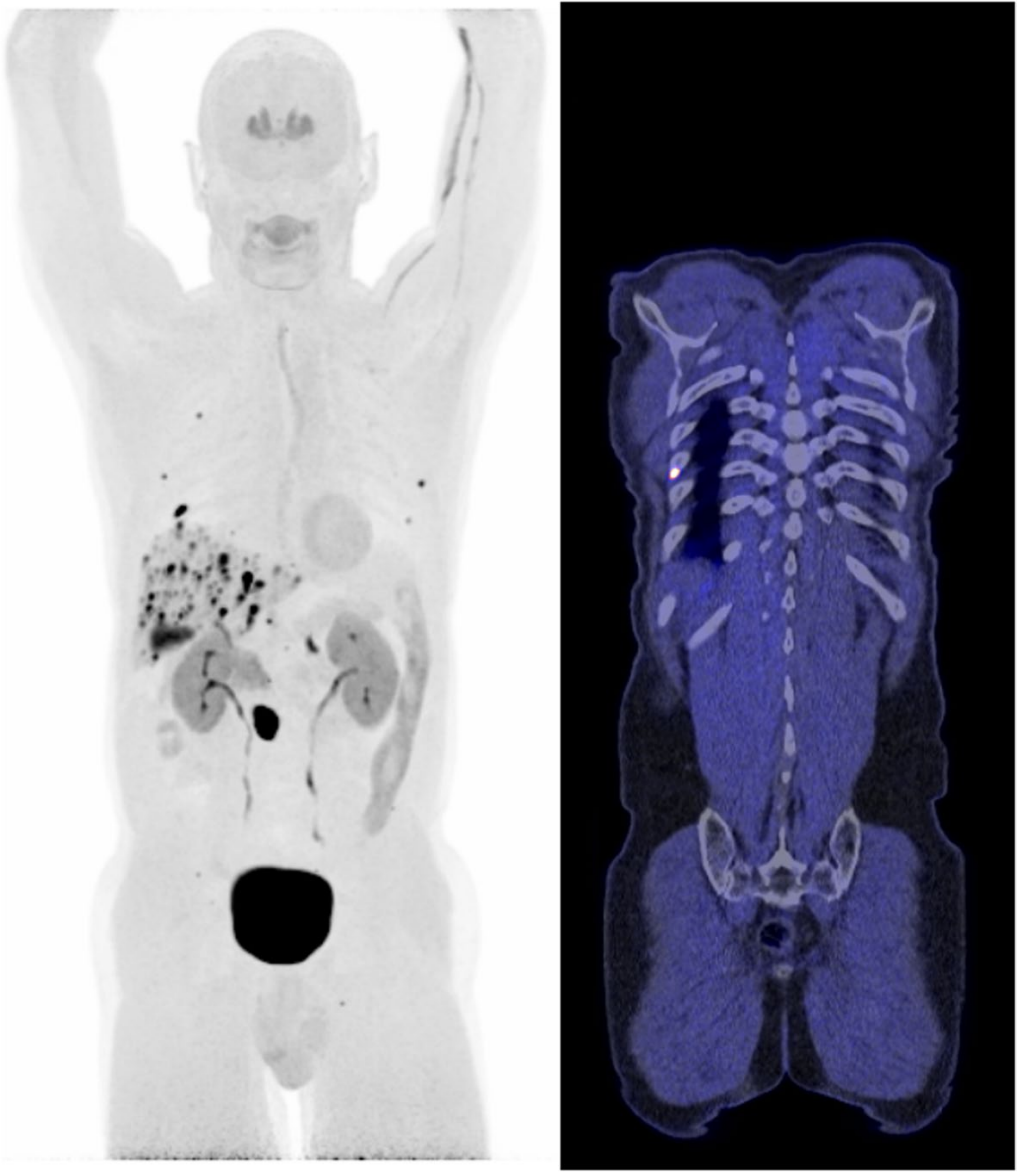
Ten minutes illustrative example of ^18^F-FDOPA LAFOV PET/CT images of a 65-year-old male patient with a neuro-endocrine tumor visualizing a mesenteric lesion, multiple liver metastases, and several bone metastases located in the ribs

**Fig. 4:**
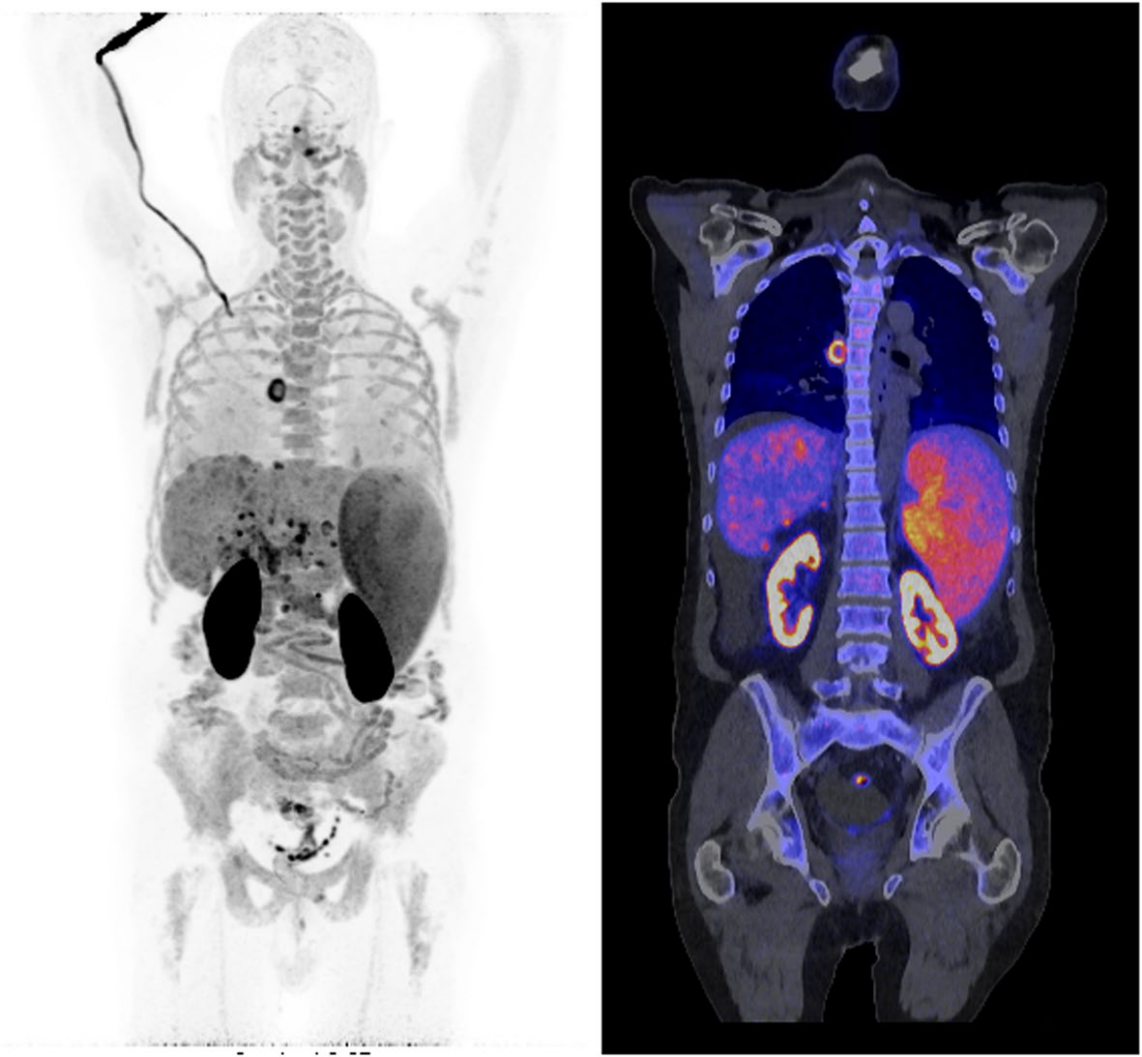
Ten minutes illustrative example of a ^11^C-Choline LAFOV PET/CT of a 68-year-old male patient with chronic hepatitis B, hepatic cirrhosis, splenomegaly, and known hepatocellular carcinoma (HCC) for which ablation therapy was given. The images show suspected recurrence of the HCC as well as multiple mesenteric, retroperitoneal, and mediastinal lymph nodes, and multiple focal uptake spots in the pelvis

For non ^18^F-FDG tracers the benefits may be larger. For ^18^F-FES, used to evaluate estrogen receptor expression in patients with metastasized breast cancer, improved sensitivity may lead to a better differentiation between low and high ER expression within a single tumor lesion. Use of LAFOV may lead to improved image quality and better signal-to-noise ratios for the ^68^Ga-labelled tracers, as generally lower amounts of activity are inject for these types of tracers. Improved signal-to-noise ratio also holds true for ^18^F-FDOPA (Fig. 3) and ^68^Ga-DOTATATE for imaging of neuro-endocrine tumors. Regarding ^11^C-Choline PET, using highly sensitive LAFOV PET allows to acquire whole body data in a single bed position. LAFOV prevents dealing with decay of the tracer in consecutively acquired bed positions influencing count statistics per step-and-shoot because of the short half-life as would be the case in conventional FOV scanners; all data is acquired simultaneously in one single bed-position (Fig. 4). ^11^C-Choline PET could be helpful in detecting hepatocellular carcinomas as these are known to frequently exhibit low ^18^F-FDG accumulation [32]. As stated earlier, immunoPET imaging with e.g., long-lived radiotracers such as ^89^Zr-labeled mAbs will be an area in which the substantial increase in sensitivity leads to a substantial improvement in image quality using LAFOV PET/CT scanners in the oncological setting [20]; enabling further development regarding labeling of mAbs beyond primarily the oncological research setting.

### Infection/inflammation

^18^F-FDG PET/CT is widely used for diagnosis and therapy evaluation in a variety of infectious and inflammatory diseases. Both infectious and inflammatory tissues actively take up ^18^F-FDG, and fungal and bacterial cells use ^18^F-FDG for their own metabolism. In addition, inflammatory mediators may also cause a local upregulation of glucose transporters [33]. The diagnostic accuracy of ^18^F-FDG PET/CT in this setting is high, but important issues to solve still remain.

First of all, the relative non-specificity of ^18^F-FDG is a major problem, and differentiation between tumor activity, inflammation and infection is not possible. Dynamic imaging with all the major organs in the field of view of an LAFOV PET/CT system, may solve this problem. As the distribution of ^18^F-FDG throughout the different organs and towards the different lesions is a dynamic process, differences in glucose metabolism may be more apparent in dynamic imaging than in routine static images one hour after the administration of ^18^F-FDG.

Secondly, for some indications imaging at later time points may be beneficial to have a better ratio between the inflammatory lesion and blood pool activity, for example in large vessel vasculitis or cardiac sarcoidosis. Since the improved sensitivity of an LAFOV PET/CT scanner allows for scanning even after 4 or 5 half-lives, this may be a worthwhile option.

Thirdly, low-grade chronic infectious processes, processes characterized by low bacterial load, and biofilms on prosthetic material, are hard to detect on the conventional FOV PET/CT systems, due to the limited sensitivity and low uptake. The increased sensitivity may enable a better detection of small and low-grade ^18^F-FDG avid foci. It may also help in the detection of smaller inflamed vessels such as in medium-sized vasculitis or inflamed cranial vessels in cranial large vessel vasculitis.

**Fig. 5:**
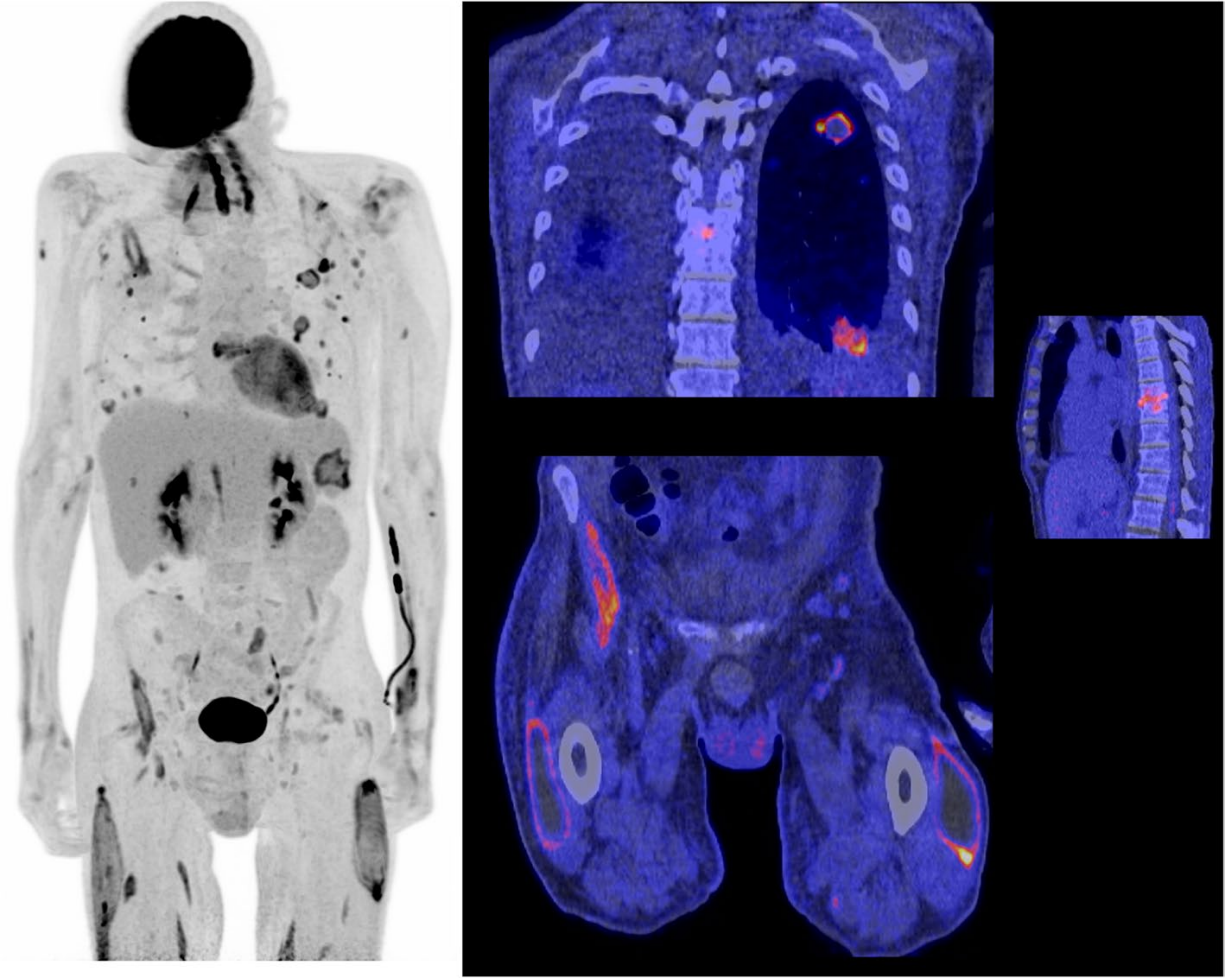
Three min illustrative example of a ^18^F-FDG LAFOV PET/CT scan of a critically ill 46 year-old male patient with methicillin-resistant staphylococcus aureus bacteremia. The acquired images show lung infections, spondylodiscitis, and abscesses in the upper legs, hip, and chest region

Last but not least, ultrafast imaging may allow for imaging critically ill patients (Fig. 5) and patients admitted to the ICU with persistent inflammation or infection. In addition, it allows for scanning children without sedation. This will allow for more flexibility in hospital planning and increase patient capacity.

### Cardiovascular

For cardiovascular imaging, LAFOV PET/CT imaging holds several advantages as well. The shorter scan duration increases the accessibility of PET/CT for patients that cannot remain supine for an extended time due to e.g., orthopnea or hemodynamic impairment. The potential to reduce administered activity could also improve the cost-benefit balance for performing baseline scans to facilitate the evaluation of intracardiac prostheses showing reactive ^18^F-FDG uptake. This may render ^18^F-FDG PET/CT at later timepoints difficult to interpret, i.e., in settings of suspected infection. Examples of this are Bentall protheses and left ventricular assist devices. Evidence is currently limited to case studies, but baseline scans have shown promise in suspected LVAD infections [34], Reactive ^18^F-FDG uptake, frequently seen in Bentall prostheses would make these interesting targets for this approach as well [35]. Other specific advantages of LAFOV PET are the possibility to performing dynamic scans, which may facilitate differentiating between reactive ^18^F-FDG uptake and uptake due to e.g. vasculitis or infective processes, and cardiac motion correction which could provide more accurate visualization of mobile structures in the heart, e.g., vegetations in suspected endocarditis frequently are missed on conventional PET/CT systems [36].

### Neurology

In a road map to implementation and especially new possibilities of LAFOV PET/CT scanners Slart et al. [37] already pointed out that brain imaging might enable combined assessment of brain and spinal cord, providing a more comprehensive assessment of the molecular basis of neurodegenerative diseases [37]. In addition, imaging of organ-axis interactions may be facilitated by these systems. This was already shown to be relevant for the brain-gut axis in Parkinson’s disease and for the cardiac-brain axis, as the latter connects cardiovascular function, neurochemical asymmetries and depression [37]. While these studies already take advantage of additional information inherent to the large axial field of view, dynamic imaging capabilities obtained from different organs and regions simultaneously further strengthen the opportunities for less invasive absolute brain quantification, first in a research setting and possibly also in a clinical setting, and for more detailed translational research of the aforementioned organ-axis interactions.

Increased sensitivity of the LAFOV systems using specific tracers further may allow for exploring involvement of previously undetectable and/or unrecognized brain regions in several neuropsychiatric disorders, while the versatility of the systems allows for lower radiation exposure or shorter scanning times, enabling brain imaging of previously more vulnerable or difficult to examine patient groups, such as children, intensive care patients or patients in general suffering from movement disorders, psychiatric pathology or claustrophobia.

The most common sites of primary cancer which metastasize to the brain are lung, breast, colon, kidney and skin cancers. Although some metastases may give rise to a wide variety of symptoms, such as headache, ataxia, seizures or paresthesia already at a very early stage, others may remain more silent for a long time. On the other hand early detection and recognition of brain metastases may have a significant impact on treatment strategies and/or prognosis.

Using ^18^F-FDG PET/CT in 2502 patients with solid extracranial neoplasms, a routine whole body ^18^F-FDG PET/ CT scan in the absence of symptoms detected brain metastasis in 1% percent of the patients when brain was included in the scan protocol [38]. The authors concluded that while on the one hand whole body PET/CT cannot replace routine imaging techniques, on the other hand positive findings provide early and crucial information for patient management, especially in asymptomatic patients [38]. It should be noted that this conclusion was drawn based on the most commonly used tracer in cancer stratification, i.e. ^18^F-FDG, for which tumor to background contrast ratio, and hence detectability, may be hindered because of the high physiological background uptake of FDG in the brain. Interestingly, in contrast to ^18^ F-FDG, a new promising candidate for tumor diagnosis, therapy stratification and follow-up, the fibroblastic activation protein inhibitor (FAPI), either labeled with ^68^Ga or ^18^F, shows negligible background activity in the brain, resulting in In higher tumor to background ratios for brain metastases from gastric, breast, lung and liver cancers, and with a higher detection rate than for ^18^F-FDG [39].

The term “chemo-brain” is sometimes used to denote deficits in neuropsychological functioning, including difficulties with memory, attention, and other aspects of cognitive function, that may occur as a result of cancer treatment consisting of chemo- or systemic therapy. In the future, systematic PET imaging (using ^18^F-FDG or other radiopharmaceuticals) for oncological stratification and follow-up may, at least in theory, provide in better understanding of this poorly understood syndrome as a basis for example for prevention, treatment or prognostication.

Finally, novel probes for imaging of translocated protein and somatostatin receptor overexpression to assess immune system reactions appear to be of additional clinical value for radiation and therapy monitoring [40]. Although from a perspective of combined brain imaging, TSPO and somatostatin tracers may be more limited with regard to their clinical application, immunoPET tracers showing tumor dissemination and load, as well as inter- and intra-tumoral expression and heterogeneity should have large clinical potential in predicting on an individual basis the most (cost) effective treatment regimens (precision medicine). With regard to the latter, several immunoPET studies already have demonstrated the detection of additional brain metastases, suggesting that even when using these tracers, patients may benefit from an LAFOV window that enables simultaneous brain imaging.

### Organ axes

It has become clear that many diseases and conditions, originally thought to be confined to a single organ, are much more complex, being involved in a cross-talk between organs, and with other organ systems [41]. Cardiorenal syndrome is defined as acute kidney injury caused by acute cardiac dysfunction such as acute decompensated heart failure and acute coronary syndrome. Deteriorating renal function can further complicate cardiac dysfunction resulting in a downward trend.

The brain–heart axis is implicated in post-stroke cardiovascular complications known as the stroke-heart syndrome, sudden cardiac death and the Takotsubo syndrome, amongst other neurocardiogenic syndromes. Dynamic ^15^O-H_2_O PET brain imaging can identify the central nervous pathways of angina pectoris, highlighting the interplay between the brain and the heart in such patients [42]. There is also evidence that connects cardiovascular function, neurochemical asymmetries and depression [43]. An ^18^F-FDG PET/CT study has linked resting amygdalar activity with cardiovascular events, indicating a potential mechanism to predict risk of cardiovascular disease caused by stress [44].

Another example is the gut–brain axis. Bacteria in the gut could have profound effects on the brain, and might be tied to a whole family of disorders [45]. There is also evidence that gut microbiota and their metabolites interfere with the host’s immune and endocrine systems [46].

Using LAFOV PET/CT systems, organ interactions can be studied before and also during therapy. Again, a better understanding of these interactions may lead to precision medicine for individual patients.

## Opportunities for artificial intelligence (AI)

The increased sensitivity and the large coverage of LAFOV systems means that number of photons originating from the body are registered by the PET detectors of the scanner. This, in turn, this results in enormous “raw” datasets. Part of this extra information translates into improved image quality as described above, but also a lot of information is not utilized during conventional image reconstruction. Storing the raw data can cause significant challenges in a hospital environment, as the datasets can be up to 1 Tb per scan depending on the tracer type, injected dose and, of course, overall scan duration. This requires high performance storage hardware such as a PB RAID array in order to prevent that these datasets need to be transferred over traditional hospital IT networking systems. However, when datasets are stored, they can provide a wealth of additional information that can be extracted using both conventional methods and artificial intelligence (AI). AI is expected to play an increasingly critical part of imaging equipment reconstruction and post-processing pipelines in the field of nuclear medicine [47].

A good example of the use of AI is given by a study of Ma et al., who showed that a deep learning reconstruction algorithm using raw LAFOV Quadra PET data as input had the potential to speed up image reconstruction and improve image quality without additional CT images [48]. Another example is a study by Sari et al., who used a deep-learning based framework to generate whole body attenuation maps on an LAFOV PET scanner by only using the system’s own lutetium-based (LSO) scintillator background radiation [49]. This would enable CT-free attenuation and scatter correction on LAFOV systems.

In summary, in the (near) future, applying AI-based methods to the wealth of data produced by LAFOV PET/CT systems can help in improving image quality and quantification and even reduce the reliance on CT-based information (thereby reducing overall radiation exposure) for corrections.

## Hurdles to overcome

Considering all the advantages mentioned above, one might think that buying and installing an LAFOV PET/CT system is a must, leading to lower administered activities, scanning new indications, new patient groups, scanning faster leading to a higher patient throughput. However, the last item is a big issue and does not simply come the purchase of an LAFOV PET/CT scanner. Several prerequisites have to be met [37], such as a radiochemistry department that is able to produce the needed amount of radiopharmaceuticals and the need of an infrastructure to allow for of rapid successive injections. This requires investment in production capacity, e.g., the need for an onsite cyclotron and the need for a laboratory which is fully automated according to Good Manufacturing Practice. Another prerequisite is the need for an update and extension of the patient facilities. More preparation rooms, waiting rooms, and changing rooms are necessary to inject and scan substantially more patients. Besides, investments are necessary for additional personnel, for the production, scanning, and reporting part, to keep up with the associated patient logistics. Furthermore, to fully explore all the possibilities of LAFOV PET/CT scanners, and to be cost effective, work hours may have to be extended, which also requires more personnel and may demand for working in shifts. Ideally, this has to be anticipated before purchasing and installing a LAFOV PET/CT scanner in a department.

## Conclusion

This review paper aimed to provide an overview of the clinical opportunities and applications for clinical practice. Apart from improved image quality and lesion detectability with respect to conventional FOV PET/CT systems, LAFOV allows e.g., reduction in acquisition times, reduction in amount of radiotracer administration, but also delayed imaging to follow tracers including labelled mAbs in vivo over an extended period of time. Furthermore, the larger axial FOV allows simultaneous investigation of the functional crosstalk between organ systems as well as continuous dynamic PET imaging of all relevant organ structures simultaneously to map pharmacokinetic behavior of (new) tracers. The future holds many opportunities for optimizing existing clinical applications using LAFOV PET, for example with the development and application of AI-based methods, and many more that have yet to be explored and introduced.

## Data Availability

Not applicable, review paper.
